# Causal link between thyroid function and schizophrenia: a two-sample Mendelian randomization study

**DOI:** 10.1007/s10654-023-01034-z

**Published:** 2023-08-17

**Authors:** Dennis Freuer, Christa Meisinger

**Affiliations:** Epidemiology, Medical Faculty, University of Augsburg, Augsburg, Germany

**Keywords:** Mendelian randomization, Thyroid function, Thyroid-stimulating hormone, Hypothyroidism, Hyperthyroidism, Free thyroxine, Schizophrenia

## Abstract

Schizophrenia is a chronic psychiatric disorder with inconsistent behavioral and cognitive abnormalities with profound effects on the individual and the society. Individuals with schizophrenia have altered thyroid function, but results from observational studies are conflicting. To date, it remains unclear whether and in which direction there is a causal relationship between thyroid function and schizophrenia. To investigate causal paths, a bidirectional two-sample Mendelian randomization (MR) study was conducted using summary statistics from genome-wide association studies including up to 330,132 Europeans. Thyroid function was described by the normal-range thyroid-stimulating hormone (TSH) and free thyroxine levels as well as an increased and decreased TSH status. The iterative radial inverse-variance weighted approach with modified second order weights was used as the main method. Based on a discovery and replication sample for schizophrenia, pooled effect estimates were derived using a fixed-effect meta-analysis. Robustness of results was assessed using both a range of pleiotropy robust methods and a network analysis that clustered genetic instruments potentially responsible for horizontal pleiotropy. Genetic liability for hypothyroidism was inversely associated with schizophrenia ($\beta =-0.06$; 95% CI: (-0.10; -0.02); $P=0.004$). No notable associations were observed between other thyroid parameters and schizophrenia. Furthermore, no associations could be detected in the reverse direction. Our results suggest that an elevated level of TSH reduce the risk for schizophrenia. The role of thyroid function and the hypothalamic-pituitary-thyroid axis in the development of schizophrenia should be subject of further research.

**Supplementary Information:**

The online version contains supplementary material available at 10.1007/s10654-023-01034-z.

## Background

Schizophrenia is a chronic psychiatric disorder with inconsistent behavioral and cognitive abnormalities [1]. Based on conservative criteria, the prevalence of the disease is 0.7% but if more comprehensive diagnostic criteria are applied, it may be higher [2]. The disease is not only associated with profound effects on the individual but also has consequences for society. Patients with schizophrenia show an impaired dopaminergic function, which is manifested in the acute psychotic state by an increase in dopamine synthesis, dopamine release, and resting-state synaptic dopamine concentrations [3, 4]. Imaging studies of the brain have shown that there is a subtle, almost universal decrease in gray matter, enlargement of the ventricles, and focal changes in white matter tracts in sufferers [5, 6]. The etiology of the disease is multifactorial and involves genetic and environmental factors [7–11]. In addition, immunological theories related to the aetiopathogenesis of schizophrenia have been proposed in recent years [12, 13]. Although considerable progress has been made in the study of schizophrenia in the last two decades, the causes of this disease are not yet fully understood [1].

There is accumulating evidence that individuals with schizophrenia have altered thyroid function, but results from observational studies do not show a consistent picture [14, 15]. In addition, little is known about the role of thyroid hormones in the pathophysiology of schizophrenia and it is not yet clear whether the close relationships between thyroid function and schizophrenia are causal or not. In this study we performed a two-sample Mendelian randomization (MR) study to investigate the causal effects of variation in normal-range thyrotropin (TSH) and free thyroxine (FT4) levels as well as hyper- and hypothyroidism on schizophrenia. Furthermore, we conducted a reverse MR analysis to assess causal effects of schizophrenia on parameters of thyroid function.

## Methods

### Study design

The MR design investigates the causal relationship between a modifiable risk factor and an outcome based on observational data, using the random genetic assignment at conception as a natural experiment. Our two-sample MR study based on summary data from genome-wide association studies (GWASs) from different cohorts of European ancestry. Detailed information about the MR-design can be found elsewhere [16].

To obtain unbiased estimates, the MR design must satisfy three core assumptions, which we will address below. A genetic instrument has to be strongly associated with the exposure of interest (relevance assumption), must not be associated with a confounder of the exposure-outcome association (independence assumption), and must influence the outcome only through the exposure (exclusion restriction assumption).

### Study population and phenotype definition

Regarding the thyroid hormone measurements, we used summary level data from a meta-analysis GWAS including 54,288 subjects from 22 and 49,269 subjects from 19 independent cohorts for TSH and FT4, respectively [17]. Individuals taking thyroid medications or undergoing previous thyroid surgery were excluded from the analyses. European subjects who were within the cohort-specific reference range were considered for the TSH and FT4 levels, which were used as continuous phenotypes. In addition, they served as a control group to which both increased (hypothyroidism) and decreased TSH levels (hyperthyroidism) exceeding the upper and lower cohort-specific reference ranges, respectively, were compared [17]. Specifically, for TSH, we used data from a more recent GWAS that expanded the considered cohort by participants from the HUNT study and the MGI repository [18]. Since this study, which included up to 119,715 subjects, did not provided summary statistics for FT4 levels, hypothyroidism, or hyperthyroidism, we used the data in replication analyses.

Two datasets were available for schizophrenia. As the discovery sample we used data from the Psychiatric Genomics Consortium (PGC) (wave 3) including 39,910 cases and 60,558 controls of European ancestry. As the PGC specializes in psychiatric phenotypes, the data are expected to be of the highest quality. In replication analyses we used summary level data from the FinnGen study (8th release) with 6,280 cases and 330,132 controls [19].

### Instrument selection

In order to fulfill the relevance assumption, SNPs (single nucleotide polymorphisms) associated with the respective exposure were selected considering an imputation score greater than 0.8 and below the genome-wide significance threshold of $5\cdot {10}^{-8}$. We then applied PLINK clumping, where bi-allelic SNPs in LD with a minor allele frequency greater 0.01 were pruned within a 10,000 kb window and a clumping threshold of 0.001 based on the 1000 genomes reference panel regarding Europeans as the super-population.

### Instrument strength and statistical power

Regarding the models using TSH and FT4 levels as continuous exposures, we calculated the variance explained by genetic instruments and quantified the instrument strength by calculating the SNP-specific F-statistics, with a value of 10 or more indicating sufficient strength and thus absence of weak instrument bias. Statistical power was calculated for a sufficient range of unknown true effects [20].

### Statistical analyses

Since the independence and exclusion restriction assumptions cannot be tested, plausibility must be assessed. Thus, focusing on the evidence, our statistical analyses consisted of four steps. First, bidirectional MR analyses were performed iteratively on the discovery and replication samples. Second, a broad range of pleiotropy robust methods were applied to assess horizontal pleiotropy. Third, meta-analyses pooled estimates were derived combining the results from discovery and replication analyses. Fourth, a network analysis was performed to identify clusters of phenotypes that serve as a potential confounder factors for an exposure outcome association and removed the affected SNPs within further sensitivity analyses.

The radial regression was used iteratively as the main framework [21]. In each iteration step we performed the inverse-variance weighted (IVW) regression with modified second order weights and calculated Cochran’s and Rueckers Q-statistics using ${\alpha }_{Q}=0.01$. Combined with graphical evaluation we were able to identify in this way potential outliers and remove them for further iterations. We then compared the consistency and distortion of estimates before and after outlier removal.

Directional pleiotropy in the final models was assessed by the radial MR-Egger intercept test and widespread horizontal pleiotropy by the MR-PRESSO global test [22]. Additionally, all models were evaluated in terms of the extent of horizontal pleiotropy considering further heterogeneity statistics as well as leave-one-out analyses and assessment of SNP-exposure vs. SNP-outcome scatter plots and funnel plots. In sensitivity analyses we applied the MR-Egger, weighted median, weighted mode, MR-PRESSO, and the MR-RAPS methods to account for different patterns of heterogeneity. Regarding the robustness of the MR-Egger estimates, the NOME (No Measurement Error) assumption was tested by calculating the ${I}_{GX}^{2}$ statistic. Finally, results from the discovery and replication studies of schizophrenia were pooled within meta-analyses using IVW fixed-effect models.

In the fourth step, we applied a PhenoScanner search to identify all known phenotypes associated with the genetic instruments used in our analyses [23, 24]. We then clustered the identified phenotypes into logical topics using a graphical network visualization. In sensitivity analyses, we removed all topic-specific SNPs and compared the results for consistency. This approach is more conservative comparing to the removal of SNPs associated with only one specific phenotype.

Results are presented either on the identity or the log scale ($\text{log}\left(OR\right)$). In case of TSH and FT4 as exposures, estimates represent the associations per one unit increase in the respective inverse-normal transformed thyroid hormone measurement. Regarding the difficult interpretation, presented estimates for all binary exposures should be viewed primarily as a test for causality. Due to the 8 null hypotheses assessed in this study, a Bonferroni corrected type I error rate ${\alpha }_{Bonf}=0.00625$ was considered to overcome multiple testing issues. MR analyses were done using primarily the packages TwoSampleMR (version 0.5.6), RadialMR (version 1.0), MRPRESSO (version 1.0), Mendelian- Randomization (version 0.6.0), mr.raps (version 0.2), MVMR (version 0.3), data.table (version 1.14.2), dplyr (version 1.0.8), and ggplot2 (version 3.3.5) of the free available statistical software R (version 4.1.2; R Foundation for Statistical Computing). Graphical network analysis was done using Gephi 0.9.

## Results

### Genetic instruments

After the instrument selection procedure 10 to 80 and 141 to 146 SNPs were considered as potential instruments for the thyroid phenotypes and schizophrenia, respectively [Supplementary Table 1, Supplementary Fig. 1]. Regarding the continuous traits, genetic instruments explained 7.5% of the variance in TSH and 3.6% in FT4. The mean F-statistics of 88.6 (TSH) and 75.5 (FT4) indicated sufficient instrument strength due to the threshold of 10. Statistical power was higher in the analyses with the discovery sample than with the replication sample and achieved a power of at least 80% when the absolute true effects were expected to be larger than 0.06 (TSH) and 0.09 (FT4) in the PGC cohort or 0.12 (TSH) and 0.17 (FT4) in the FinnGen cohort [Supplementary Fig. 2].

### Main analyses

In the following, we present the results based on the radial IVW regression with modified second-order weights as the main analysis.

**Fig. 1:**
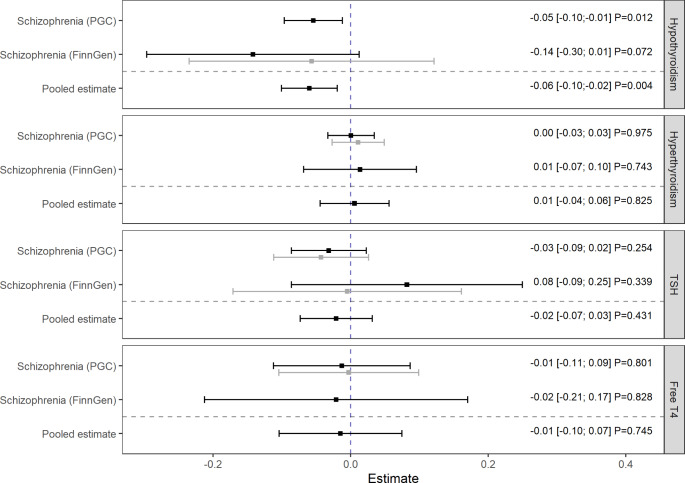
Causal estimates and 95% confidence intervals for the effect of thyroid function on schizophrenia in the PGC (discovery) and the FinnGen (replication) cohorts. Estimates obtained from the iterative radial inverse-variance weighted regression with second order weights and combined within a fixed- effect meta-analysis. Grey color represents estimates from the first and black color the estimates from the last iterations. P values should be interpreted based on the Bonferroni corrected threshold ${\alpha }_{Bonf}=0.00625$

**Fig. 2:**
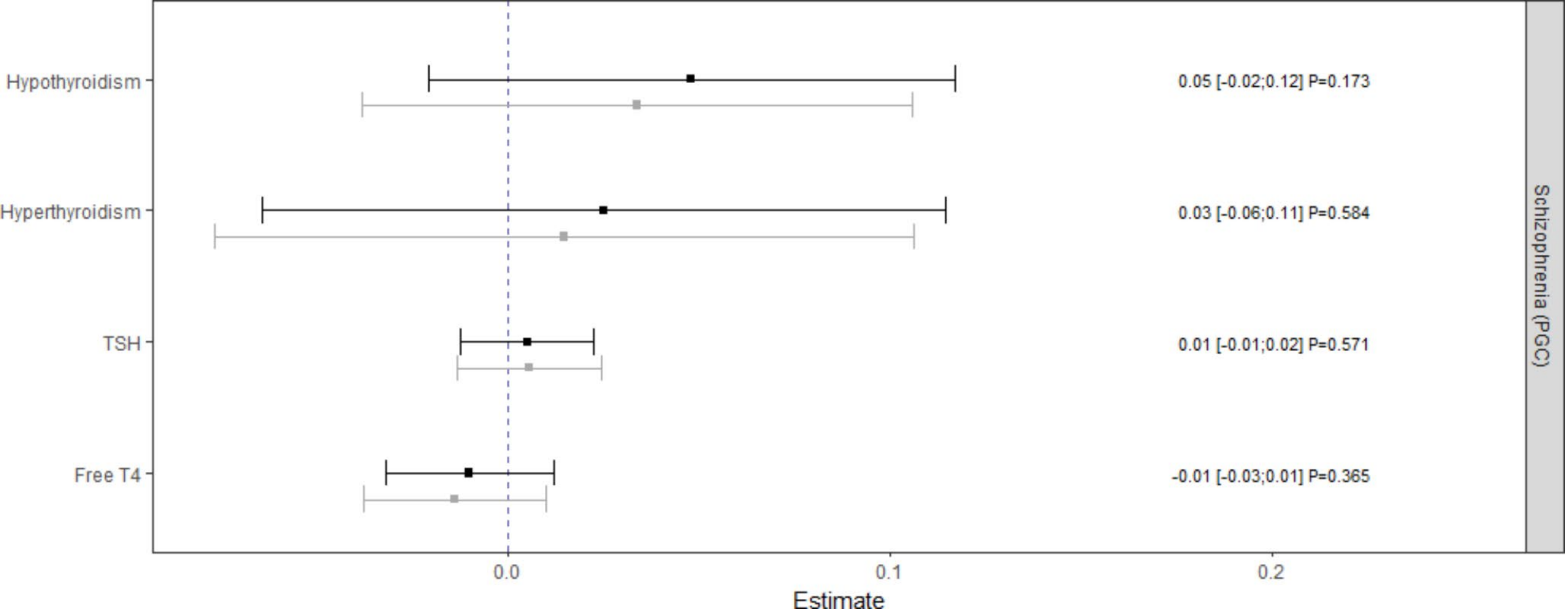
Causal estimates and 95% confidence intervals for the effect of schizophrenia on thyroid function. Estimates obtained from the radial inverse-variance weighted regression with second order weights. Grey color represents estimates from the first and black color the estimates from the last iterations. P values should be interpreted based on the Bonferroni corrected threshold ${\alpha }_{Bonf}=0.00625$

Genetically predicted hypothyroidism was inversely associated with the risk of schizophrenia [Fig. 1]. The result of the discovery analysis ($\beta =$-0.05; 95% CI: (-0.10; -0.01); $P=$0.012) was confirmed by the consistent point estimate of the replication analysis ($\beta =$-0.14; 95% CI: (-0.30; 0.01); $P=$0.072), leading to the final estimate of the meta-analysis ($\beta =$-0.06; 95% CI: (-0.10; -0.02); $P=$0.004). However, no notable associations could be found between hyperthyroidism, TSH, and free T4 with schizophrenia neither in discovery nor in replication analyses [Fig. 1, Supplementary Fig. 3]. There was also no evidence for an association between genetically predicted schizophrenia and thyroid function (reverse direction) with point estimates ranging from − 0.01 to 0.05 [Fig. 2]. Due to the lack of suitable genetic instruments, only estimates based on the PGC cohort could be calculated in the reverse direction. All results considering TSH could be fully confirmed with the replication dataset [Supplementary Figs. 4–5].

### Sensitivity analyses

Outlier removal, to the extent that it was done as part of our iterative process [Supplementary Table 2], did not substantially changed the results [Figs. 1 and 2]. Basically, all estimates based on the PGC cohort were more precisely resulting in narrower confidence intervals compared to consistent estimates based on the FinnGen cohort. After removal of outliers, which are listed in Supplementary Table 3, there was no evidence of directional pleiotropy or substantial heterogeneity for the models assessing the association between hypothyroidism and schizophrenia with respect to the radial MR-Egger intercept test, MR-PRESSO global test, and various Q-statistics [Supplementary Table 4]. However, heterogeneity could be observed modelling the effects of TSH on schizophrenia and the bidirectional models between FT4 and schizophrenia. Regarding the ${I}_{GX}^{2}$ statistics that are above 0.968, the NOME assumption can be considered valid for all models. Furthermore, all pleiotropy robust approaches confirmed an inverse effect of hypothyroidism on schizophrenia and supported largely the bidirectional results obtained by the radial IVW method [Supplementary Figs. 4 and 5].

**Fig. 3:**
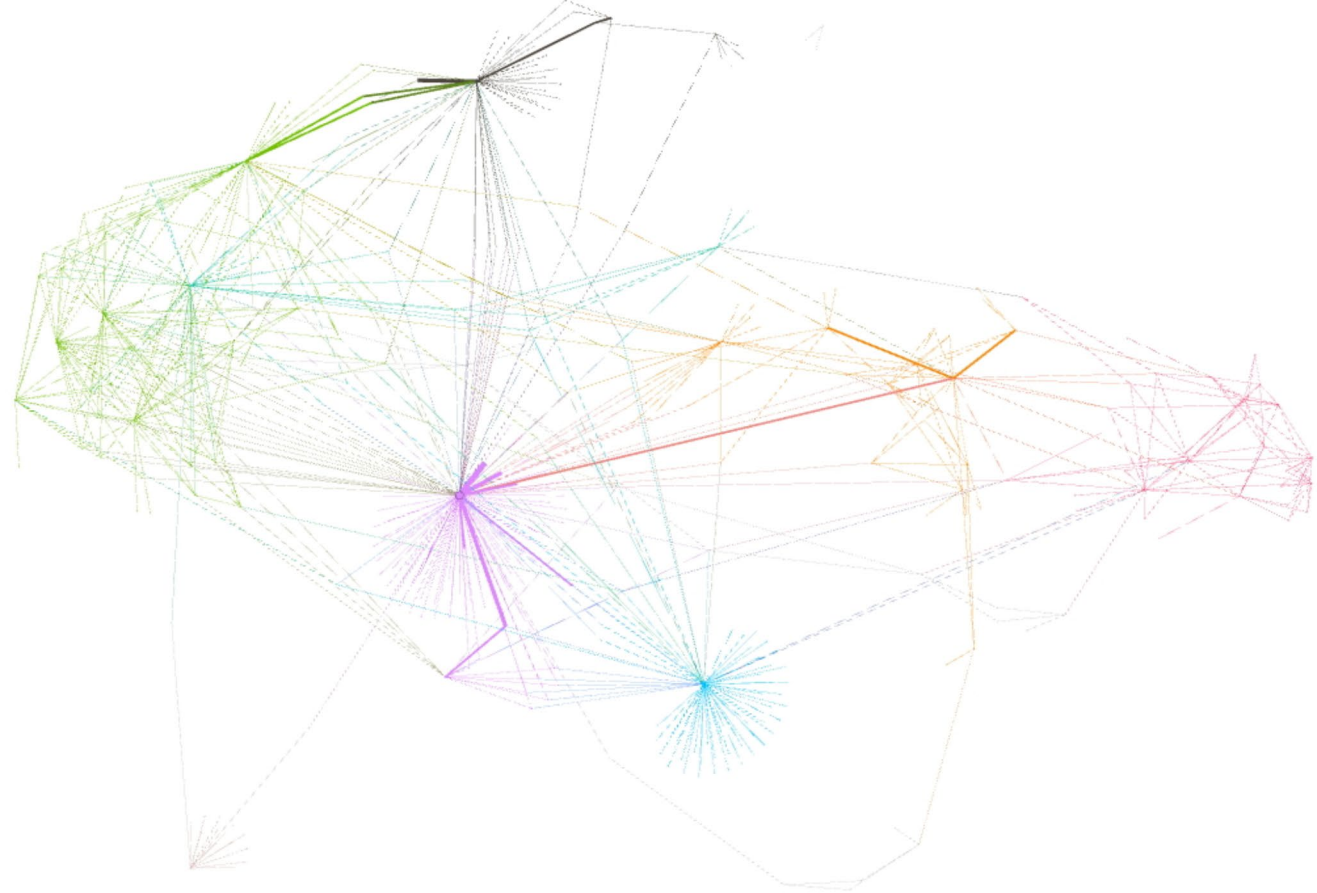
Results from a network analysis showing associations between the genetic instruments for thyroid function and all known phenotypes listed in PhenoScanner. Identified clusters of interests were allergic diseases (purple), autoimmune diseases (orange), cancer (green), cardiovascular diseases (light blue), dental health (black), diabetes (blue-green on the left side), immune system (red), lifestyle (light brown), obesity (light green), and Parkinson (blue-green on the right side)

Finally, removal of cluster-specific SNPs obtained from the network analysis [Fig. 3, Supplementary Table 5] showed consistent estimates except for the association of FT4 with schizophrenia in the FinnGen cohort [Supplementary Fig. 6]. Interestingly, removal of the multi-cluster SNP *rs597808* led to a statistically significant estimate for the association between hypothyroidism and schizophrenia in the FinnGen cohort.

In summary the inverse relationship between genetic liability to hypothyroidism and the risk for schizophrenia can be considered robust, since all different approaches and sensitivity analyses yielded similar results in different cohorts with no evidence of bias due to weak instruments or horizontal pleiotropy.

## Discussion

In this MR study we demonstrated a causal inverse association between hypothyroidism and schizophrenia, while none of the other markers of thyroid function were significantly associated with the outcome. In the reverse direction no significant associations could be found.

Our findings are contrary to a cross-sectional population-based study from Israel, which compared the percentage of schizophrenia between hypothyroid patients (n = 40,843) and age and sex frequency-matched healthy controls (n=40,918). It was found that the proportion of schizophrenia was significantly higher in patients with hypothyroidism than in controls and multivariable logistic regression models confirmed an independently positive association between hypothyroidism and schizophrenia (OR = 1.62; 95% CI: (1.45; 1.82)) [16]. A recent population-based study observed an increased rate of hypothyroidism in patients with schizophrenia after, but not before, the onset of the disease [15]. Another community-based study including 1252 patients with schizophrenia and 3756 controls reported no difference regarding the distribution of TSH levels between the groups [15]. However, after initiation of antipsychotic treatment patients with schizophrenia suffered more often from hypothyroidism than control subjects. Thus, it could be possible that antipsychotics may affect thyroid hormone levels [25], but whether these drugs have a direct impact on the hypothalamic pituitary-thyroid axis is largely unknown yet [26]. A recent systematic review and meta-analysis including 19 studies reported that the levels of TSH might be decreased in patients with first episode psychosis and increased in those with multiple-episode psychosis [26]. Other studies observed that hyperthyroidism may manifest with psychotic symptoms in patients, while hypothyroidism may present with mood problems similar to the negative symptoms of schizophrenia [27, 28]. Overall, the literature to date on this topic provides very different results. In addition, there are no longitudinal studies investigating the associations between thyroid hormones and the development of psychosis [26]. Furthermore, studies on the role of antipsychotic drugs in this context are still missing [26].

Thyroid hormones influence neurodevelopmental processes such as proliferation, migration, differentiation, and synapse formation of neurons [29]. In adults, they interact with glial cells that modulate immune responses, regulate neurotransmitter release, and control neuron metabolism [30]. Thyroid hormones play a role in modulating dopaminergic, serotonergic, glutamatergic, and GABAergic networks [31]. The central neurotransmitters dopamine, norepinephrine, and serotonin interact with hypothalamic regulatory hormones in controlling anterior pituitary function. Dopamine, in particular, has been associated with inhibitory control of TSH secretion, since a decrease in circulating TSH has been observed after administration of dopamine [32, 33] or dopamine agonists [34, 35]. In addition, an increase in serum TSH in response to the specific dopamine receptor blocker drug metoclopramide in patients with primary thyroid insufficiency was found [36]. But also the serotonergic andglutamatergic system, which are involved in schizophrenia are linked to thyroid hormones [31]. Thus, a causal relationship between hypothyroidism or elevated TSH levels and the manifestation of schizophrenia seems plausible, although the exact pathophysiological causes have not yet been clarified.

The present study is the first two-sample MR on the causal association between thyroid function and schizophrenia. Our findings might be of clinical significance as they showed reliable results by employing various MR methods. In addition, the presented results seem not to be affected by pleiotropy, because the estimates obtained in the sensitivity analyses were consistent. Heterogeneity was minimized by a more conservative iterative implementation of the radial regression framework with modified second-order weights, which improved outlier detection and removal as well as heterogeneity assessment compared to the usual approach. Furthermore, consistency of point estimates could be confirmed before and after removal of outliers by this method. However, there are also several limitations. The findings of the study were based on data from individuals of European descent and replicated by using data from the FinnGen study. Notwithstanding the presence of population stratification, i.e. the presence of some genetic differences between individuals from northern European countries compared to individuals of other European origins, the results also appear to be generalizable to “other subpopulations”. Whether the results can be transferred to non-European ethnicities have to be investigated in further studies. A further shortcoming of the study is,that we were unable to differentiate between first episode and multiple-episode psychosis [26].

In conclusion, the present investigation found an inverse relationship between hypothyroidism and schizophrenia. The results underscore the relevance of the thyroid function in the manifestation of schizophrenia. Research on the role of the hypothalamic-pituitary-thyroid axis in the development of schizophrenia should be subject of further research.

### Electronic supplementary material

Below is the link to the electronic supplementary material.

Supplementary Material 1: Figure S1 Flowchart of the genetic variant selection process. Figure S2 Power calculations. Figure S3 Meta-analyses based on the discovery and replication MR studies. Figure S4 Sensitivity analyses investigating the effect of thyroid function on schizophrenia. Figure S5 Sensitivity analyses investigating the effect of schizophrenia on thyroid function. Figure S6 Sensitivity analyses based on network clustering results. Table S1 Genetic variants used as instruments in the bidirectional Mendelian randomization analyses. Table S2 Genetic variants used as instruments before and after outlier-exclusion. Table S3 Identified pleiotropic SNPs within bidirectional Mendelian randomization analyses. Table S4 Heterogeneity statistics. Table S5 Results from PhenoScanner search.

## Data Availability

The present study is based on freely available summary statistics from genome-wide association studies. Data regarding thyroid function can be obtained from https://transfer.sysepi.medizin.uni-greifswald.de/thyroidomics/datasets/. Summary-level data for schizophrenia used as the discovery and replication sample can be found at https://www.med.unc.edu/pgc/download-results/ and https://www.finngen.fi/en/access_results, respectively.

## List of Abbreviations

CI: Conficence interval
FT4: Free Thyroxine
GWAS: Genome-wide association study
IVW: Inverse variance weighted
MR: Mendelian randomization
NOME: No measurement Error
OR: Odds ratio
PGC: Psychiatric Genomics Consortium
SNP: Single nucleotide polymorphism
TSH: Thyrotropin
